# Clustering swap prediction for image-text pre-training

**DOI:** 10.1038/s41598-024-60832-x

**Published:** 2024-05-24

**Authors:** Sun Fayou, Hea Choon Ngo, Yong Wee Sek, Zuqiang Meng

**Affiliations:** 1 Guangxi University, Nanning, 530004 Guangxi China; 2 Huzhou College, Huzhou, 313000 Zhejiang China; 3 Linyi University, Linyi, 276000 Shandong China; 4 Universiti Teknikal Malaysia Melaka, 76100 Durian Tunggal, Melaka, Malaysia

**Keywords:** Model pre-training, Clustering learning, Swap prediction, Cluster number, Computer science, Information technology

## Abstract

It is essential to delve into the strategy of multimodal model pre-training, which is an obvious impact on downstream tasks. Currently, clustering learning has achieved noteworthy benefits in multiple methods. However, due to the availability of open image-text pairs, it is challenging for multimodal with clustering learning. In this paper, we propose an approach that utilizes clustering swap prediction strategy to learn image-text clustering embedding space by interaction prediction between image and text features. Unlike existing models with clustering learning, our method (Clus) allows for an open number of clusters for web-scale alt-text data. Furthermore, in order to train the image and text encoders efficiently, we introduce distillation learning approach and evaluate the performance of the image-encoder in downstream visual tasks. In addition, Clus is pre-trained end-to-end by using large-scale image-text pairs. Specifically, both text and image serve as ground truth for swap prediction, enabling effective representation learning. Concurrently, extensive experiments demonstrate that Clus achieves state-of-the-art performance on multiple downstream fine-tuning and zero-shot tasks (i.e., Image-Text Retrieval, VQA, NLVR^2^, Image Captioning, Object Detection, and Semantic Segmentation).

## Introduction

The zero-shot approaches are reasonable in practice use. Meanwhile, a lot of methods (e.g., VLMo^1^, Coca^2^, BEiT-3^3^, etc.) proved that image-text fusion can obviously improve the performance of feature representation. In view of this, this paper deeply explores a novel image-text cluster fusion method to achieve progress on a broad range of downstream tasks. As is known to all, the supervised learning methods can only be applied to a range of categories, which are difficult to achieve zero-shot inference^4^. In recent years, multimodal methods can learn image feature representations from image-text pairs, which achieved great success (e.g., image classification^5^, object detection^6^, semantic segmentation^7^, image captioning^60–62^, VQA^63^, cross-modal retrieval^64^, etc.). Currently, due to the diversity of downstream tasks, researchers use different parts of a pre-trained model to adapt to various tasks by building blocks^3,8^. Furthermore, community solutions focus on encoder-only or encoder-decoder manner^9^.

CLIP^4^ used open-vocabulary as labels with encoder-only manner to unseal an era of zero-shot computer vision tasks, which has inspired many influential researches in the recent past. Currently, CLIP is effective during performing some tasks (e.g., image classification, image-text retrieval, etc.). Meanwhile, the inferiority of CLIP is that modal intersection only uses a simple cosine similarity, which performs poorly in tasks with complex modal interactions (e.g., visual reasoning, etc.). However, cross- modal feature deep fusion is profitable^10^. To solve above problem, researchers proposed lots of methods (e.g., VLMO^1^, BEiT-3^3^, etc.) that only use a transform encoder for multimodal fusion. Specially, these methods support different experts to handle different types of input (i.e., text, image), known as MoME (mixture of multi-expert). In addition, lots of multimodal models adopt cross-attention method for modal fusion and an encoder produces the final outputs^13,14^. Although these methods are very powerful and achieve promised performance, there are unable to efficiently perform generative tasks (e.g., image captioning).

On the other hand, researchers design encoder-decoder architecture for generative tasks. Notably, the decoder fuses feature, which are from image and text encoders, and the decoder auto-regressively generates feature representation^8,15^. However, some methods (e.g., SimVlm^12^, OFA^16^, etc.) are less efficient due to the lack of text-only representation of image embedding alignment. To address this issue, CoCa^2^ adopts image-text comparative (ITC) loss for cross-modal alignment before multimodal fusion, which obviously improves inference performance. Currently, image-text alignment is popular in most multimodal models. Another typical method is BLIP^11^, which utilizes 2 text encoders, 1 text decoder and an image feature encoder to calculate 3 loss function for different downstream tasks. However, although encoder-decoder manner performs well in generative tasks, it performs inconspicuously in generic tasks^10^ (e.g., image classification, object detection, etc.).

Inspired by above approaches, we discuss a question: is it possible to deep mine image-text fusion to learn a high-performance image-text model for most downstream tasks? In order to achieve this objective, this paper utilizes a lot of tricks. In fact, our idea draws on previous research: (1) distillation learning can enhance generalization, (2) image-text alignment can capture the correlation between features, (3) cluster center with explicit semantics, (4) swap prediction is beneficial for the consistency of image and text features. Specifically, we use V-FFN and L-FFN^3^ as teachers to train image and text encoders and then carry out image-text alignment by comparative learning manner. Furthermore, this paper utilizes swap prediction method to produce image-text feature cluster prototype, which can evidently improve robustness and performance. Finally, we employ LongNET^20^ as a cross-attention module to fuse long-sequence input tokens and generates features for downstream tasks.

**Figure 1:**
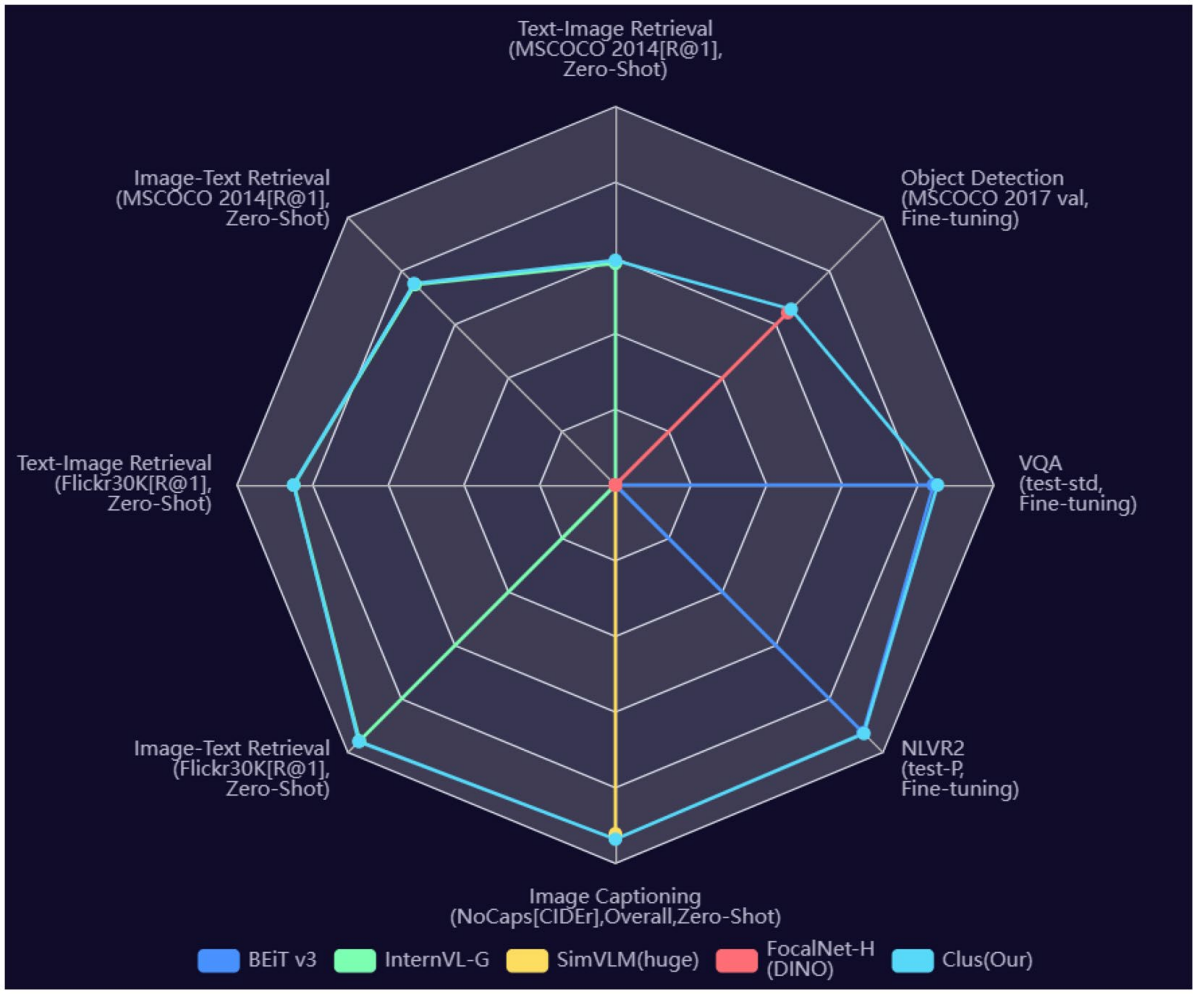
Performance illustration of Clus. Our method achieves the promised results.

All in all, this paper adopts encoder-only architecture and multiple tricks (e.g., distillation learning, cluster learning, swap prediction, etc.) to achieve benefits on lots of downstream tasks. The performance of our method (Clus) is illustrated in Fig. 1. Our contributions are summarized as follows:

1. This study fills the gap of cluster learning for large-scale multimodal model pre-training.
2. Swap prediction is beneficial to improve the explicit semantics of each clustering center.
3. Our method (Clus) achieves the SOTA performance on downstream tasks and proves that the generic large-scale image-text model is useful for practical tasks.

## Related work

In this section, we review the existing visual-language approaches, which are related to this research. To overcome the challenging for our objective, this section discusses image and text encoders, image-text fusion and cluster learning.

### Image-encoder and text-encoder

Specifically, image and text encoders are pre-trained separately that is a widely used method. As is known to all, image-encoder plays an important role in vision-language (VL) tasks. Currently, the prevalent methods for image-encoder are ViT-based or distillation learning manner. CLIP^4^, LSeg^17^, CLIPasso^18^, ActionCLIP^19^, et al. used ViT-based manner to train image-encoder on different datasets. The ablation studies proved that the performance of the network has obviously improved when image-encoder employed some tricks. Similarly, ViLD^21^ trains image-encoder by distillation learning, which sped up the training and generalization ability of the network. Thus, visual-embed module needs to be a complex network architecture. However, these two manners are independent at present.

Relatively speaking, text-encoder network has a simple architecture and high maturity. Specifically, BERT^22^ is a typical method for language model (LM) pre-training and fine-tuning on downstream tasks. At the same time, ALBEF^14^ et al. adopt ViT as the text-encoder, which uses 50% of transformer layers as the text-encoder and other layers as the image-text fusion module. This approach improves performance by image-text alignment and multimodal fusion. However, this method requires a lot of computing cost. Recently, VLMo^1^ used well pre-trained vision expert (V-FFN) and multi-head self-attention module to train language expert (L-FFN) to achieve SOTA performance.

In view of this, we adopt distillation learning and the parameters of ALBEF^14^ for initialization image and text encoders to improve the training efficiency.

### Image-text fusion

**Figure 2:**
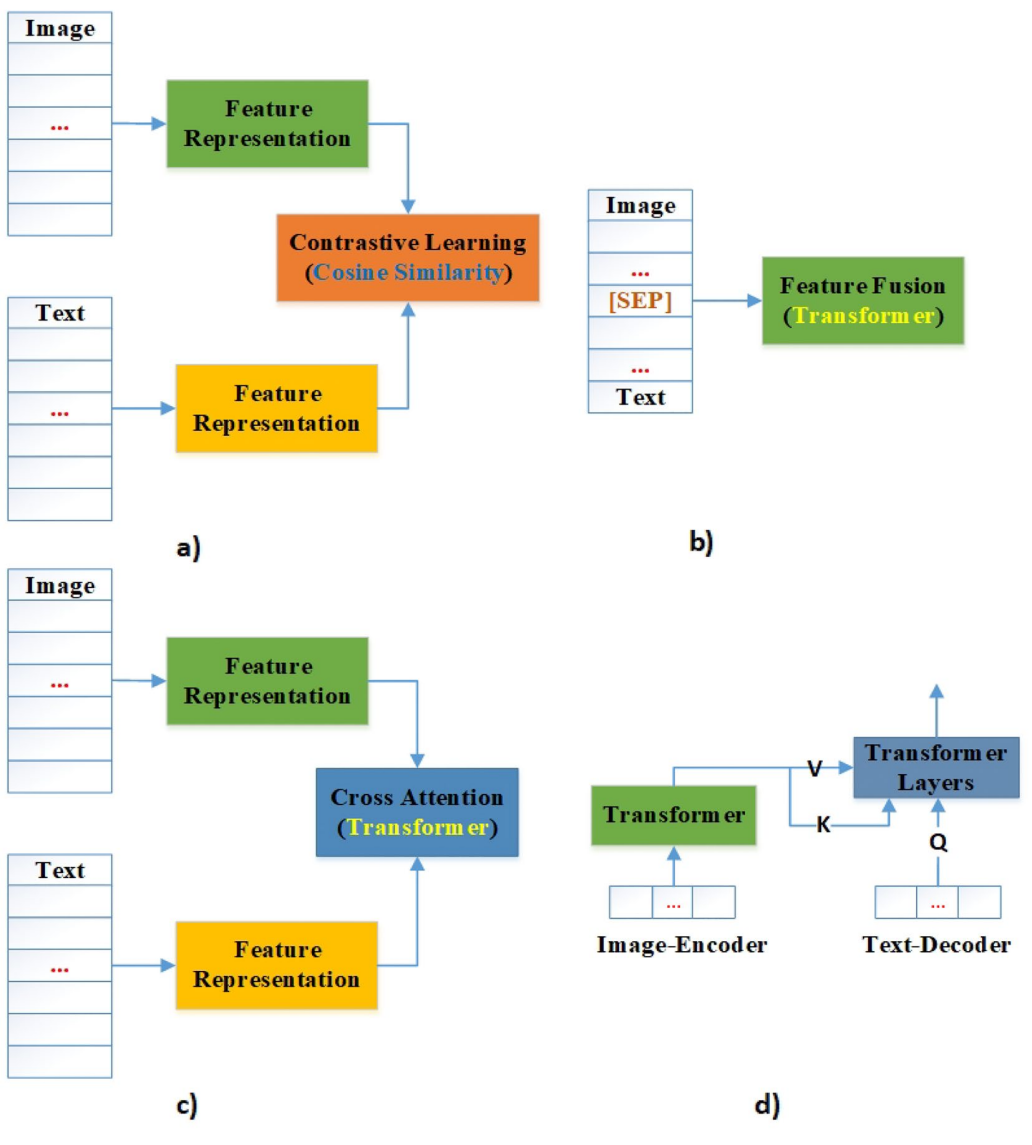
Illustration of the four architecture types.

Intuitively, single modal cannot achieve clear and accurate feature representation. Currently, multimodal feature fusion methods can obtain a more comprehensive and reasonable representation. In order to better utilize the features of various modalities, researchers need to consider the correlation and weight between different modalities. Nowadays, there are four manners for image-text fusion, as shown in Fig. 2.

The 1st method is similarity-based (Fig. 2a), which is contrastive learning manner. However, due to the simplistic process of modal interaction, these methods are ordinary in performance for difficult tasks (e.g., VQA, Visual Reasoning, etc.). Later, researchers were aware of the important role of modal interaction for multimodal learning. Therefore, the community adopted networks with efficient representation ability to replace cosine similarity for modal interaction. The 2nd method (e.g., VisualBERT^13^, UNITER^15^, ViLT^23^, etc.) is transformer-based multimodal fusion (Fig. 2b), which is single-stream method where the two modalities are concatenated and separated by a special token (e.g., [SEP]). This approach achieves unconstrained multi-modal fusion. The 3rd method (e.g., ALBEF^14^, CoCa^2^, etc.) is dual-stream (Fig. 2c), where the image and text features are first processed by two independent transformer layers, and then all features are fed into multimodal fusion module (e.g., transformer layers, etc.). Dual-stream method explicitly constrains interactions between modalities and effectively introduces an inductive bias in each model, but it also introduces additional parameters. The 4th method (e.g., BLIP^11^) is based on the encoder-decoder architecture (Fig. 2d), which is a good generative model (e.g., image captioning, etc.). Typically, encoder utilizes multi-head self-attention to fuse inputs from the encoder and decoder. Currently, single-stream methods and dual-stream methods have their own superiority in different downstream tasks. Thus, it is hard to draw a conclusion. In view of this, the dual-stream approach is adopted according to our model architecture in this study.

### Cluster learning

Cluster learning is used to construct the meaningful cluster center from unlabeled datasets. Currently, researchers use it to produce better discriminative feature representations.

Currently, cluster Learning methods are widely used in computer vision tasks. ClusDet^24^ unifies object cluster and detection. ORE^25^ adopts contrastive clustering and unknown-aware proposal network for Object Detection. Specially, Contrastive Learning methods also used cluster Learning. GroupViT^8^ integrates grouping blocks in transformer layers as image-encoder for semantic segmentation. SwAV^26^ uses "swapped" prediction manner to compare image features. PiCIE^27^ uses invariance and equivariance in Clustering for unsupervised semantic segmentation. HAIS^28^ introduces the hierarchical aggregation to makes full use of spatial relation of points and point sets for 3D Instance Segmentation. In view of this, this study adopts cluster learning to improve the consistency between modalities.

## Method

This section introduces the proposed Clus which contains four modules, i.e., image and text encoders, multimodal fusion block, image-text clustering, and reasoning.

**Figure 3:**
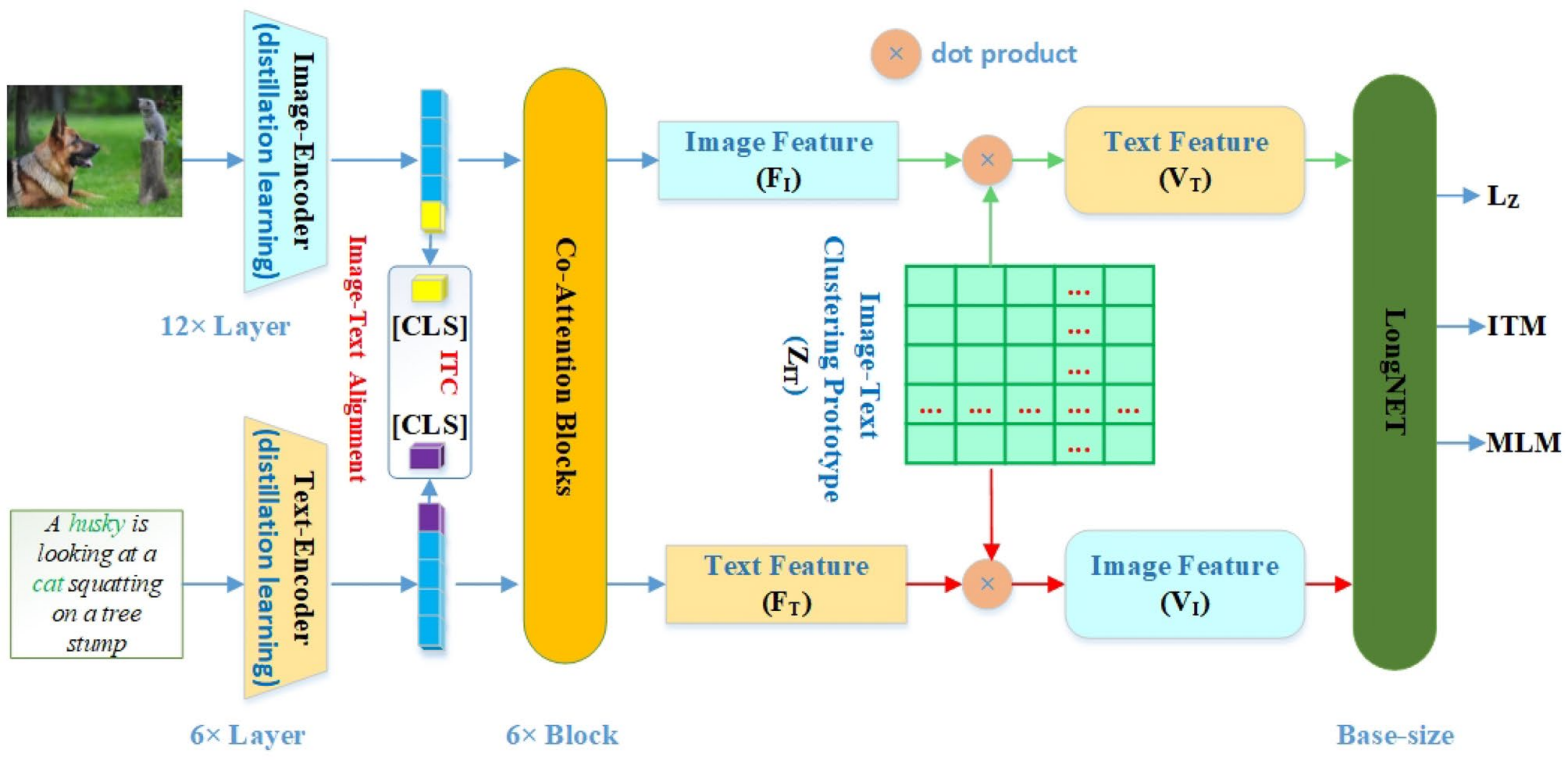
An overview of the Clus. Image and text encoders are distilled sub-networks. Image-text alignment adopts lower-dimensional [CLS] tokens to solve the unimodal representations. There is deep cross-modality middle-fusion with 6 co-attention blocks. The clustering prototype vector (Z_IT_) is a trainable image-text pair feature vector, which is trained with cross-prediction method. In addition, L_Z_ is the clustering swap prediction loss. Especially, Clus is trained in an end-to-end way. Finally, the loss function is ITC (image-text contrastive learning loss) + ITM (image-text matching loss) + MLM (masked language modeling loss) + L_Z_. In addition, ITM adopts global hard negative mining^3^ method.

The architecture of Clus is shown in Fig. 3. From Fig. 3, it can be observed that Clus consist of distillation learning block, co-attention block, clustering swap prediction block, and LongNET block. Specially, ViT is the backbone of encoders that are trained by distillation learning manner. Then, co-attention blocks are used for image-text feature fusion. Subsequently, we adopt clustering swap prediction method to achieve cross-prediction of image feature (V_I_) and text features (V_T_). Finally, the large number of feature vectors from the cluster are brought into LongNET^20^. Moreover, ITM loss and LM loss are used to achieve optimal matching and prediction. Note that the image-text alignment is the same as ALBEF^14^, where the dimension of [CLS] tokens is R^256×1^.

Note that BEiT-3^3^, VLMo^1^, etc., treat images as a foreign language and adopts mask strategy for pre-training. Specially, an image is split into non-overlapping patches and then some patches are randomly masked. Although these methods achieved the SOTA performance at the time, this strategy harms the local neighboring structures especially when discriminative regions are split. To alleviate this issue, this paper employs unmasked images and achieves SOTA performance by strategy of clustering swap prediction. Specially, due to the huge cluster number, we adopt LongNET^20^ that scales token length to 1 billion with linear computational cost.

### Image and text encoders

**Figure 4:**
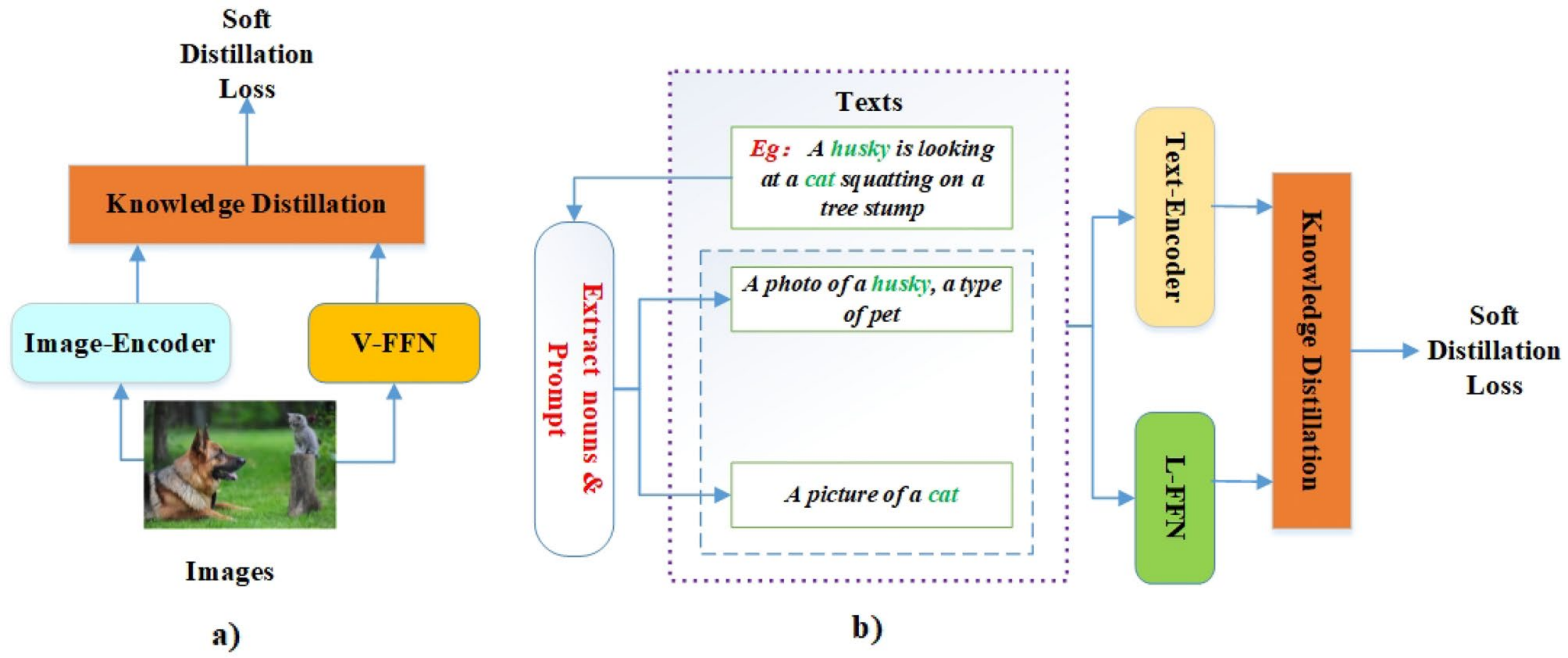
The distillation learning illustration of image-encoder (**a**)) and text-encoder (**b**)). The natural language toolkit (NLTK) is adopted for extracting nouns. Specifically, the encoders are trained together with the network, and the trained encoders are used for reasoning.

As is known to all, knowledge distillation enables the network with novel semantic representations for downstream tasks. Nowadays, V-FFN and L-FFN^3^ have good performance for vision and text feature representation respectively in open-vocabulary tasks. Thus, this study uses them as teachers, as shown in Fig. 4. At the same time, in order to improve the training efficiency and performance, the encoders are initialized with the parameters of ALBEF^14^. In a word, distillation learning enables our model (Clus) to be general and energy-efficient. Specifically, this study adopts soft distillation loss. The loss function consists of Kullback–Leibler divergence loss (soft loss) and cross entropy loss (hard loss) are as follows:

1 $${\mathbf{Loss}} = {\upalpha L}_{{{\rm soft}}} + (1 - {\upalpha )L}_{{{\rm hard}}}$$

where $${\upalpha = 0}{.45}$$.

As shown in Fig. 4b, this study adopts the “Prompt Template” method to produce extra text labels for each image in addition to the original sentence label. In other words, k nouns are randomly selected from a sentence, and every noun word is prompt with a set of handcrafted prompt templates. Specifically, there are many templates for downstream tasks (e.g., object detection, VQA, etc.). The motivation is that objects in images are more likely to be described by nouns, and it is beneficial for supervised labels.

### Multimodal fusion

**Figure 5:**
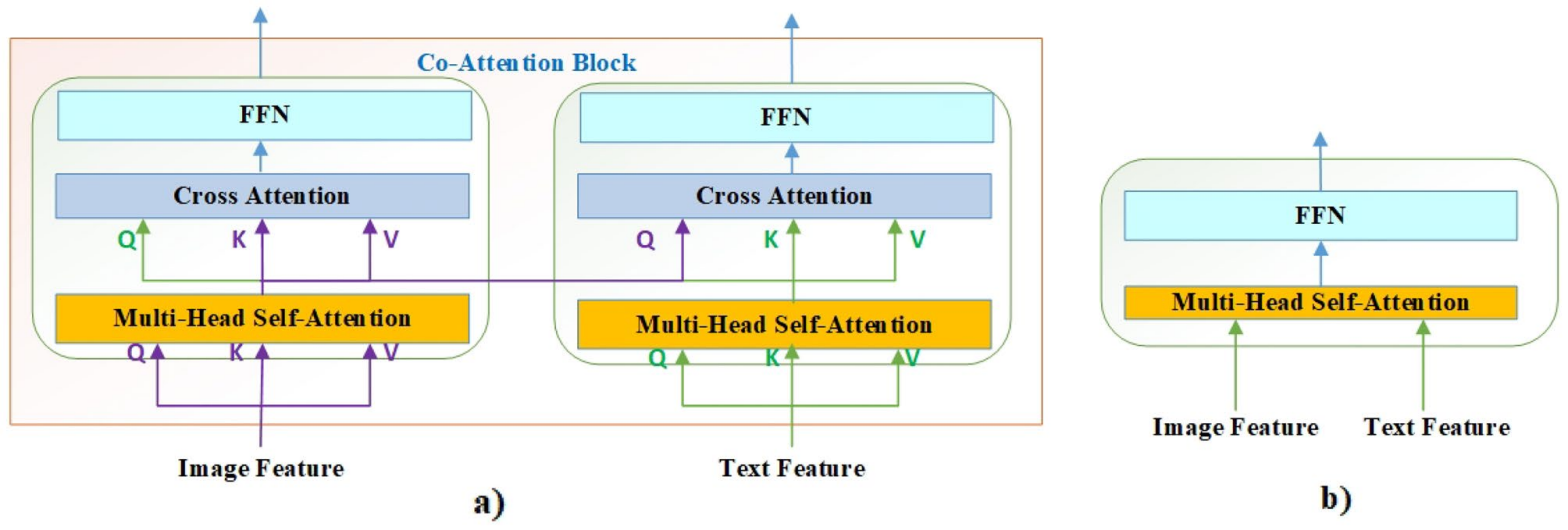
Illustration of two types of multimodal fusion modules: (**a**) co-attention, and (**b**) self-attention.

Clus studies two multimodal fusion methods and investigates their performance, as shown in Fig. 5. In the co-attention method, image and text features are fed into different encoders respectively, where each encoder consists of self-attention module, cross-attention module, and one feed-forward module. However, the self-attention method only adopts transformer encoder layer. Concurrently, compared to self-attention method, co-attention method utilizes cross-attention to achieve multimodal interaction, and image and text modalities can be transformation independently. Specially, VOLTA^29^ demonstrated that these two methods can achieve comparable performance. Thus, this study uses co-attention method to match dual-stream architecture. Furthermore, our experiments prove that co-attention performs better. In addition, this study designs 6 co-attention blocks so that the number of parameters of these two models are roughly close to each other.

2 $${\mathbf{Att(Q,K,V)}} = {\rm softmax(}\frac{{{\rm QK}^{{\rm T}} }}{{\sqrt {{\rm d}_{{\rm k}} } }}{)}$$

3 $${\mathbf{Att}}_{{\mathbf{I}}} { = (\rm Q}_{{\rm T}} {,\rm K}_{{\rm I}} {,\rm V}_{{\rm I}} {)}$$

4 $${\mathbf{Att}}_{{\mathbf{T}}} { = (\rm Q}_{{\rm I}} {,\rm K}_{{\rm T}} {,\rm V}_{{\rm T}} {)}$$

where Att_I_ and Att_T_ denote image and text cross-attention respectively, as shown in Fig. 5a. From Eqs. (3)–(4), it can be observed that cross modal feature fusion is achieved by Q-vector from different modalities.

### Image-text clustering

Currently, clustering method is effective in multimodal pre-training. SOHO^55^ and SwAV^26^ only performed an online clustering on visual feature maps, which are lack of the representation of image-text feature consistency. TL;DR^56^ used K learnable image-text embedding vectors to achieve a small, high-quality set for vision-language pre-training. However, TL; DR^56^ is not enough for zero-shot tasks with only K embedding vectors. Meanwhile, SwALIP^57^ employed swapped prediction method, yet it used representations from the other modality as prototypes. One objective of this study is to use images and text as supervised labels respectively for feature learning. Thus, we design a multimodal jointly embedding space method by online clustering manner. Simply, this study proposes an online swap prediction method that utilizes the advantages of contrastive learning without comparing the feature of image-text pair. Specifically, Clus boosts the consistency of positive samples for clustering while learning features of the image-text pairs rather than directly comparing features as the contrastive learning. Furthermore, Clus adopts "swap" manner to predict another modality representation from one modality feature and the clustering prototype vector. Specially, we use the DBSCAN^30^ method to achieve automatic clustering. Furthermore, the loss function for clustering swap prediction is as follows:

5 $${\mathbf{L}}_{{\mathbf{Z}}} { = }\,{{\rm L}({\rm P}}_{{\rm T}} {,\rm G}_{{\rm T}} {) + L(P}_{{\rm I}} {,\rm G}_{{\rm I}} {)}$$

6 $${\mathbf{V}}_{{\mathbf{T}}} { = dot(F}_{{\rm I}} {,\rm Z}_{{{\rm IT}}} {),\rm F}_{{\rm I}} \in {\rm R}^{{{i} \times {\rm d}}} {,\rm Z}_{{{\rm IT}}} \in {\rm R}^{{{\rm d} \times {\rm k}}}$$

7 $${\mathbf{V}}_{{\mathbf{I}}} { = dot(F}_{{\rm T}} {,\rm Z}_{{{\rm IT}}} {),F}_{{\rm T}} \in {\rm R}^{{{\rm t} \times {\rm d}}}$$

8 $${\mathbf{LongNET}}_{{{\mathbf{Input}}}} { = concat(V}_{{\rm T}} {,\rm V}{}_{{\rm I}}{)}^{{\rm T}}$$

where $L(V_{T} ,G_{T} )$ and $L(V_{I} ,G_{I} )$ are cross entropy loss. k is the number of cluster and Z_IT_ is clustering prototype vector in Eq. (6). The $G_{T}$ and $G_{I}$ are the ground truth of text and image respectively. Thus, this study uses $L(V_{T} ,G_{T} )$ as a case.

9 $${\mathbf{L(P}}_{{\mathbf{T}}} {\mathbf{,G}}_{{\mathbf{T}}} {\mathbf{)}} = - \sum\limits_{{\rm k}} {{\rm G}_{{\rm T}}^{{{(\rm K)}}} {\rm log}^{{{\rm P}_{{\rm T}}^{{{(\rm k)}}} }} } {,\rm P}_{{\rm T}}^{{\rm k}} \in {\rm softmax(}{\mathbf{V}}_{{\mathbf{T}}} {)}$$

Specifically, Eqs. (6)–(7) represent cross-prediction of different modalities respectively. At the same time, we use the loss function (i.e., Eq. (5)) to achieve the consistency of positive samples.

We argue that the advantages of cluster swap prediction are, (1) improve the performance of the image and text encoders, (2) each clustering center has a clear semantic, which helps to efficiently determine the positive image-text pairs, (3) the size of clustering prototype vector (Z_IT_) can be dynamically adjusted with downstream tasks, and memory cost is acceptable and not explosive due to the dimension = 2.

### Reasoning

**Figure 6:**
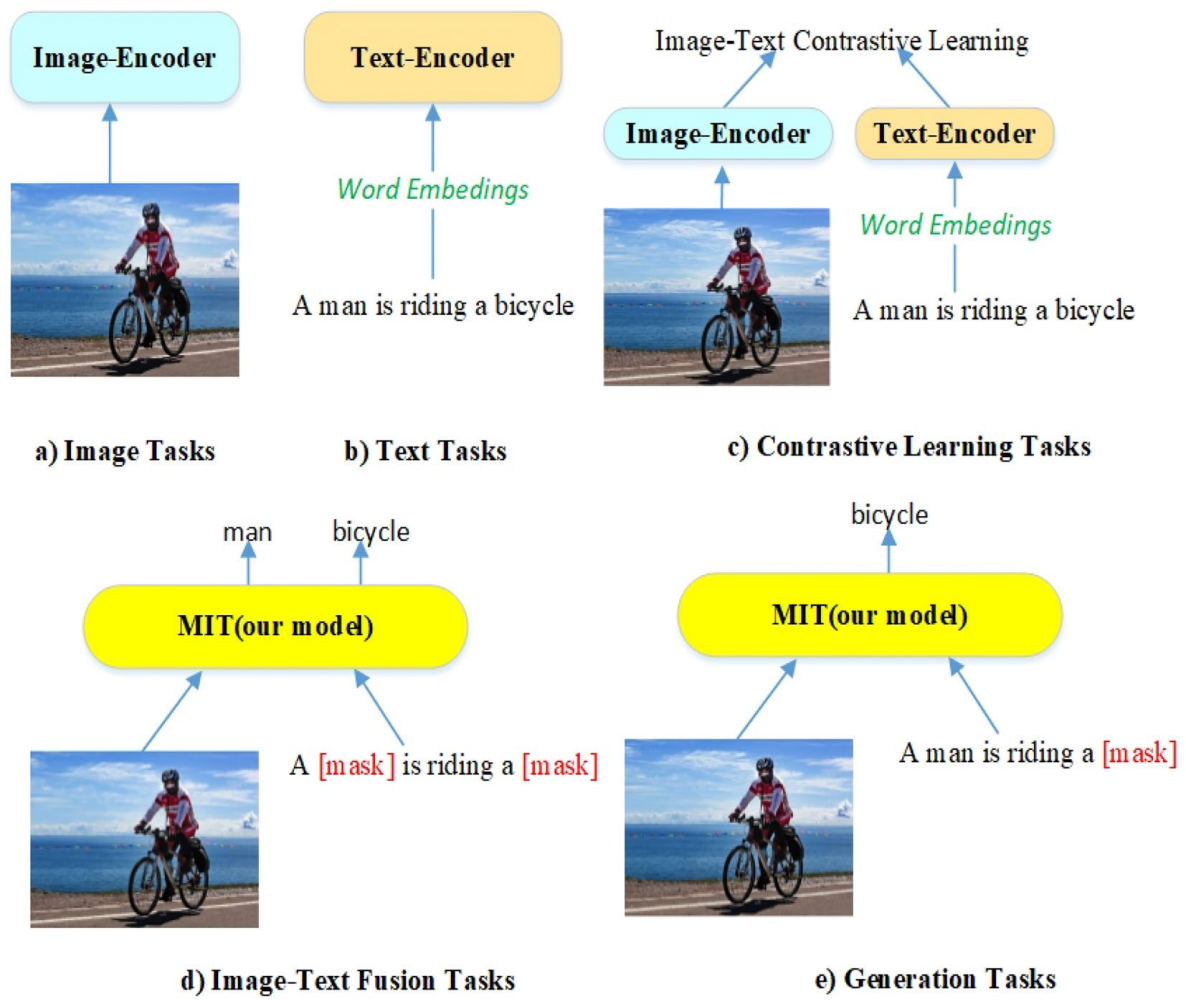
An illustration of Clus (our model) for downstream tasks.

Our another objective is to train a model with flexible architecture by end-to-end manner for downstream tasks. The advantage of flexibility can be shown in reasoning. Clus performs monomodal and multimodal downstream tasks, as shown in Fig. 6. For example, Fig. 6c is suitable for carrying out retrieval multimodal tasks. Figure 6d is masked image-text multimodal model for VQA, NLVR^2^, etc. Figure 6e masks some words during training and only images are input for reasoning, which is used for generation multimodal tasks (e.g., image captioning, etc.). Specially, our method like stacking blocks to solve architecture inconsistency issue for achieving a unified model.

### Computational complexity

The computational complexity of Clus mainly involves three parts, i.e., co-attention, clustering prototype vector, and LongNET. Specifically, Swin Transformer^59^ proposed that the computational complexity of vision transformer can be denoted as:

10 $${\mathbf{\Omega (ViT)}}{ = o(4Ld}^{{2}} { + 2L}^{{2}} {d)}$$

where L is the sequence length and d is the hidden dimension.

Due to the architecture of co-attention is ViT, its computational complexity is consistent with Swing Transformer.

11 $${\mathbf{\Omega (co - attention)}}{ = }{\mathbf{\Omega (ViT)}}$$

Meanwhile, the computational complexity of the clustering prototype vector is as follow:

12 $${\mathbf{\Omega (clustering)}}{ = o((i + t)} \times {d} \times {k) = o(Ldk)}$$

where k is the number of cluster.

In addition, it has been proved in the paper^20^ that LongNET can successfully scale up the sequence length with almost constant runtime and save memory. The computational complexity is as follow:

13 $${\mathbf{\Omega (LongNET)}}{ = o(Lk)}$$

Thus, the computational complexity of Clus is as follow:

14 $$\begin{gathered} {\mathbf{\Omega (Clus)}}{ = \Omega (co - attention) + \Omega (clustering) + \Omega (LongNET)} \hfill \\ { = o(4Ld}^{{2}} { + 2L}^{{2}} {d) + o(Ldk) + o(Lk) = o(Lk)} \hfill \\ \end{gathered}$$

where d is constant.

In view of this, the complexity of Clus is near linear manner and reasonable.

## Experiments

Nowadays, BEiT-3^3^ has achieved the promised performance, but its image-encoder, text-encoder and visual-language encoder are trained independently. Therefore, inspired by ALBEF^14^, CoCa^2^, etc., this study utilizes large-scale image-text pairs to pre-train our models with end-to-end manner. Specially, our model will be evaluated on both monomodal and multimodal downstream tasks by fine-tuning or zero-shot manner. In addition, the results of the comparison methods are from the paperswithcode.com (deadline: 8/1/2024). The url of our codes is https://github.com/dlearing/Clus.git.

### Pre-training data

In order to achieve end-to-end pre-training, we directly use large-scale image-text pairs to pre-train image-text encoders and our multimodal model. In addition, we adopt widely used web datasets, i.e., CC12M^31^, SBU captions^32^, COCO^33^, Visual Genome^34^, LAION-400M^35^ and RedCaps-12M^36^.

### Pre-training settings

Following ViT^37^, the resolution of input image is 224 × 224 and the dimension of token is 1 × 768. The batch size is 2048 image-text pairs. Furthermore, we adopt AdamW^38^ optimizer with β_1_ = 0.9, β_2_ = 0.999 and a weight decay of 0.01. Concurrently, the learning rate is warmed-up to the peak value of 1e−4 in the first 5% of training steps with a cosine schedule. In addition, we configure a learnable temperature parameter with an initial value of 0.06 in ITC loss. Specially, DBSCAN^30^ is applied to automatically generate the number of cluster centers. Finally, we employed 8 Nvidia A100 GPU 80 GB cards and spent about 6 days for pre-training.

### Downstream visual-text tasks

We demonstrate the performance of our method through 4 downstream experiments. Specially, the hyper-parameters of visual question answering (VQA), NLVR^2^, and image captioning tasks are shown in Table 1.

**Table 1:** Hyper-parameters for VQA, NLVR^2^, and image captioning tasks.

Hyper-parameters	VQAv2	NLVR^2^	Image captioning
Optimizer	AdamW		
Peak learning rate	2e−5	3.5e−5	5e−5
Fine-tuning epochs	8	10	10
Warmup epochs	1	4	1
decay schedule	Cosine schedule		
Weight decay rate	0.01	0.05	0.01
batch size	256	256	128
AdamW β_1_	1e−8		
AdamW β_2_	0.9,0.995		
Input resolution	256	384	512

### Image-text retrieval

There are two sub-tasks, i.e., image-to-text retrieval, and text-to-image retrieval. Likewise, CoCo and Flickr30K^39^ datasets are adopted for this task and the Karpathy split^40^ is used for both datasets. We conduct fine-tuning our model for 10 epochs with 1024 batch size and the input image resolution is 384 × 384. In addition, the learning rate reaches peak value 3.5e−5 within the 1st epoch by a cosine schedule. The weight decay is 0.01. The architecture of the modal is shown in Fig. 6C. The experimental results are as follows.

**Figure 7:**
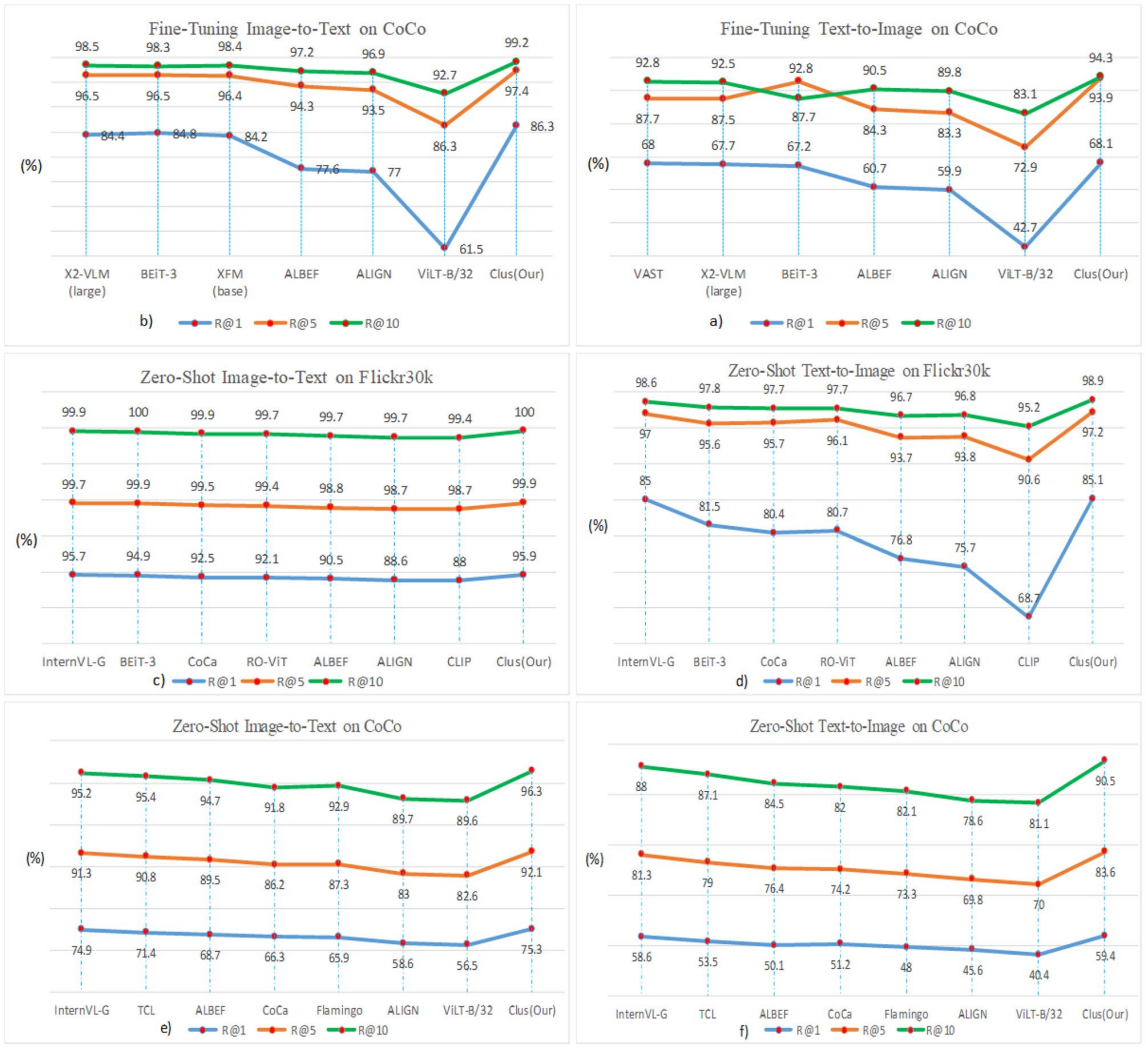
Results of image-text retrieval on COCO and Flickr30K.

From Fig. 7, it can be observed that our method gains a leading advantage in both fine-tuning and zero-shot tasks. We believe that the strategy of clustering multimodal embedding space improves the performance of the image and text encoders. Meanwhile, the performance of image-to-text retrieval tasks demonstrates that images have richer discriminative features.

### VQA and NLVR^2^

Our method is suitable for VAQ and NLVR^2^ tasks, and the strategy is shown in Fig. 6d. We think that VQA is a classification issue and fine-tune and evaluate on the VQA 2.0 dataset^41^. For the VQA task, image-question pairs are fed into network and a MLP classifier is appended for prediction. Likewise, For NLVR^2^, this study uses each triplet (one caption and two images) to construct two image-text pairs as input. The final outputs of the two pairs are concatenated and then fed into a MLP to predict the label.

**Figure 8:**
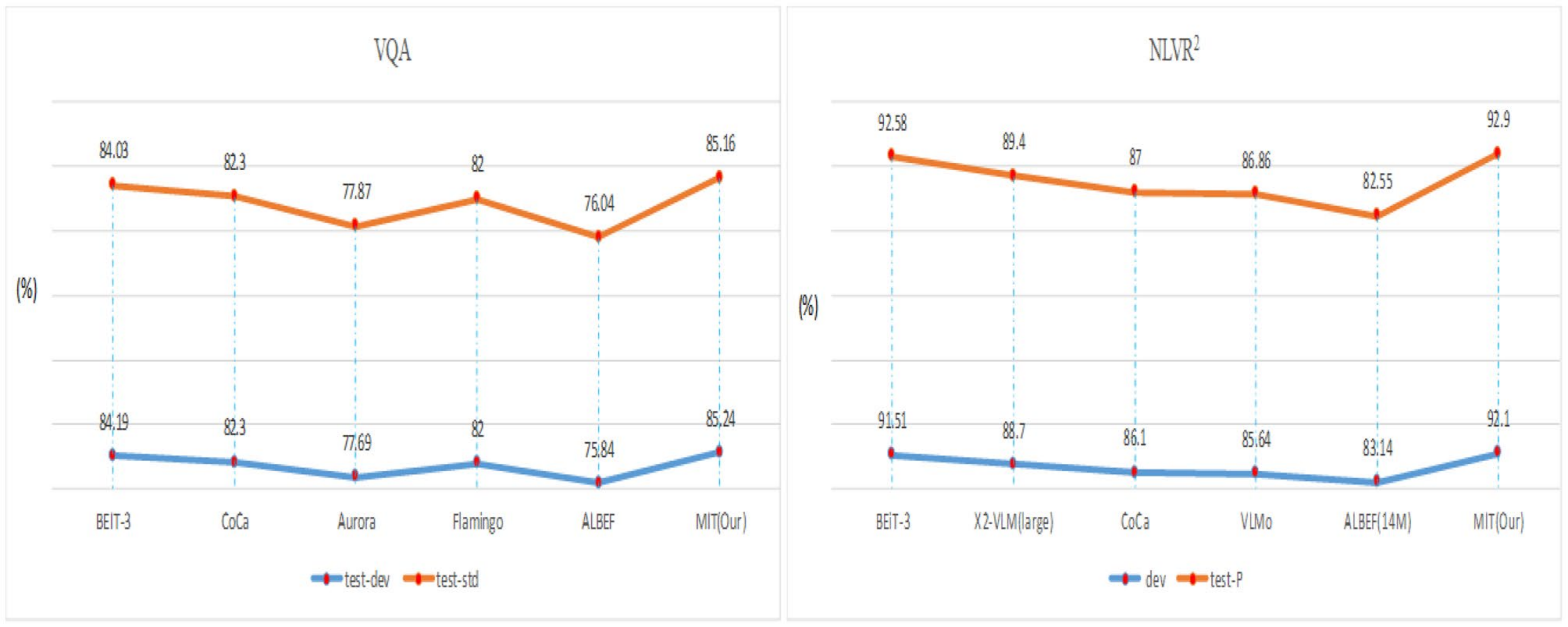
Results of VQA and NLVR^2^ tasks.

From Fig. 8, it can be observed that our method outperforms BEiT-3^3^ by 1.13 points in test-std for VQA. In addition, our model achieves 0.32% gain compared to BEiT-3 in test-P for NLVR^2^.

### Image captioning

Generative tasks are challenging that relies on the clues of image-text and text-text. Specially, we follow the previous methods (e.g., VLMO^1^, UNILM^42^) by a masked fine-tuning manner to learn clues. The strategy is shown in Fig. 6e. Meanwhile, we input image-text pairs into model and randomly mask some caption words for fine-tuning. During reasoning, we only feed images into model to generate the caption tokens in an autoregressive manner. Furthermore, we adopt language model (LM) loss without CIDEr optimization.

From Table 2, it can be observed that our model gets a further 9% improvement compared to BEiT-3 in CIDEr. Furthermore, our model outperforms previous methods in 4 metrics and achieves SOTA performance.

**Table 2:** Results of image captioning on fine-tuned COCO captioning.

Model	Spice	Meteor	BLEU-4	CIDEr
mPLUG^43^	26	32	**46.5**	155.1
OFA^16^	**26.6**	32.5	44.9	**154.9**
BEiT-3^3^	25.6	32.4	44.1	147.6
CoCa^2^	24.7	**33.9**	40.9	143.6
Clus(Our)	27.8	34.2	47.2	156.6

Significant values are in bold.

As shown in Table 3, the zero-shot performance of our model is competitive with fine-tuned model as PaLI-17B. Specially, our model outperforms PaLI-17B by 3.2% in in-domian metric and all prior methods in zero-shot. This is because In-domain contains lots of COCO Captioning data. However, Near-domain and Out-of-domain sets have a lot of strange data. Meanwhile, PaLI adopts an encoder-decoder architecture with innate advantage for image captioning. Specifically, the performance of Clus is very close to PaLI.

**Table 3:** CIDEr results of image captioning on zero-shot NoCaps caption.

Model	In-domain	Near-domain	Out-of-domain	Overall
SimVLM(base)^12^	83.2	84.1	82.5	83.5
SimVLM(huge)^12^	101.2	100.4	102.3	101.4
mPLUG^43^	86.34	81.5	90.49	84.02
BLIP-2^11^	**123.7**	120.2	124.8	121
PaLI-17B(Fine-tuning)^8^	121.1	**124.4**	**126.7**	**124.4**
Clus(Our)	124.3	122.8	125.3	123.6

Significant values are in bold.

In this task, we believe that clustering can help construct image-text clues. Concurrently, language model (LM) loss is beneficial for improving the generalization of Generative tasks.

### Vision downstream tasks

In order to demonstrate the impact of clustering-swap strategy on the performance of image-encoder, we carry on object detection and semantic segmentation experiments.

### Object detection

For a fair comparison, we pre-train image-encoder on the Object365^44^ and conduct experiments on the COCO2017^33^ benchmark. we adopt our image-encoder as backbone and use the strategy of ViTDet^45^ for object detection. Likewise, Soft-NMS^46^ is employed for reasoning.

**Figure 9:**
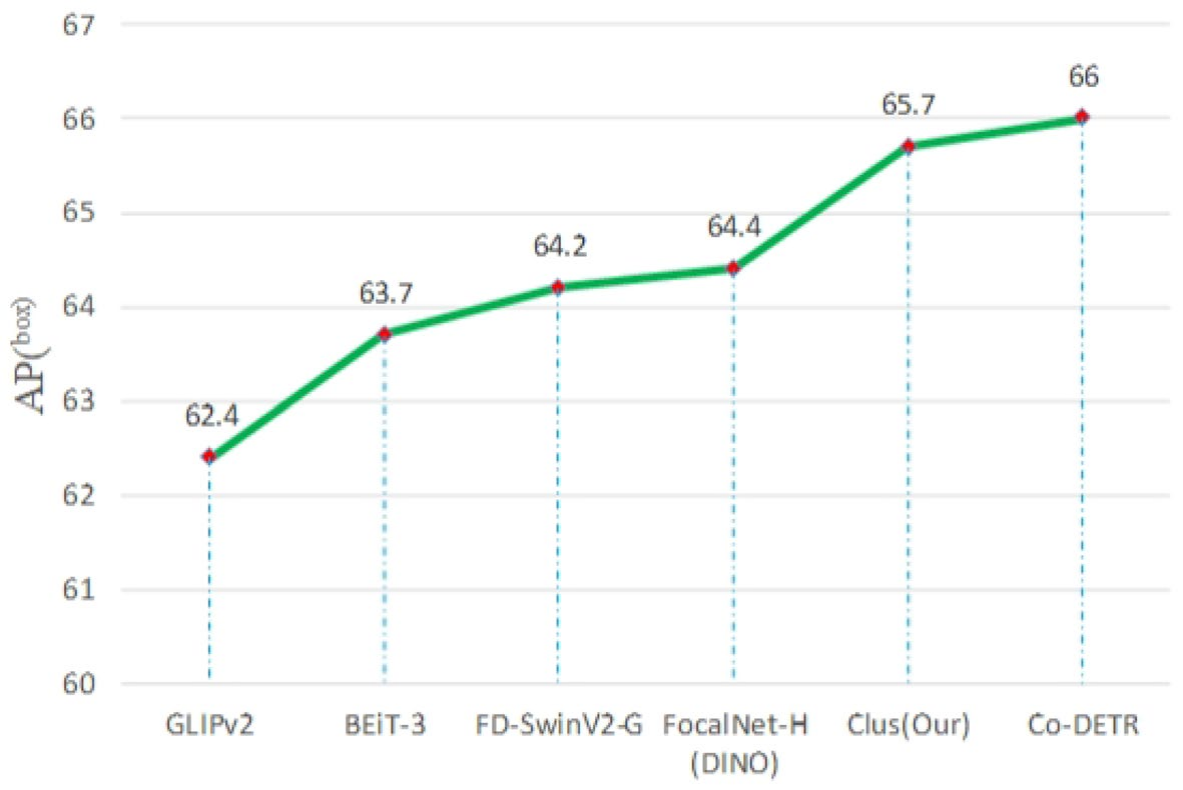
Results of object detection on COCO2017.

From Fig. 9, it can be observed that the AP of image-encoder is improved by 1.3% compared with FocalNet-H (DINO)^47^ and is only 0.3% lower than the SOTA supervised model Co-DETR^48^. Ultimately, it proves that clustering swap is beneficial for encoder training.

### Semantic segmentation

Generally, natural image pre-trained models are hard to achieve amazing results in medical image segmentation. Notably, the scarcity of public available medical imaging data affects the progress of medical models. In view of this, we transfer the parameters of our image-encoding to SAM-Md3D^49^ model and validate the performance on the BraTS2021 dataset. The input resolution is 240 × 240 × 155. Specially, we use AdamW^38^ optimizer with β_1_ = 0.9, β_2_ = 0.98 and weight decay is 0.01. The learning rate is warmed-up to 4e−3 within 10 epochs and decayed to 2e−3 following a cosine schedule. In addition, the loss is the combination of dice loss and cross-entropy loss.

**Figure 10:**
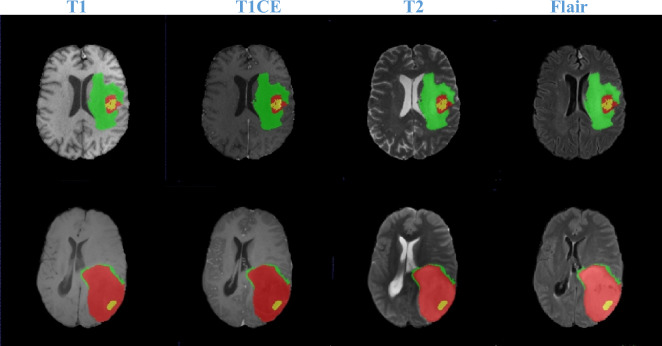
The semantic segmentation results of four sequences for Brain Tumors. The yellow is enhancing tumor regions. Tumor core is composed of yellow and red regions. The green, yellow and red consist of whole tumor.

From Table 4, it can be observed that the model using the parameters of our image-encoder is significant advantages in 3 tests (i.e., WT, ET, TC). Specially, it brings 3% AVG gains compared to Swin UNETR^53^. Therefore, we believe that natural images pre-trained models are beneficial for improving the performance of other tasks. Notably, Fig. 10 shows the segmentation results of four sequences.

**Table 4:** fivefold cross validation for dice metrics.

Model	Whole tumor	Enhancing tumor	Tumor core	Avg
nnU-Net^50^	0.929	0.88	0.917	0.909
SegResNet^51^	0.93	0.878	0.912	0.906
TransBTS^52^	0.915	0.867	0.893	0.892
Swin UNETR^53^	0.933	0.891	0.917	0.914
Image-encoder (our)	0.951	0.934	0.946	0.944

### Image-text clusters

**Figure 11:**
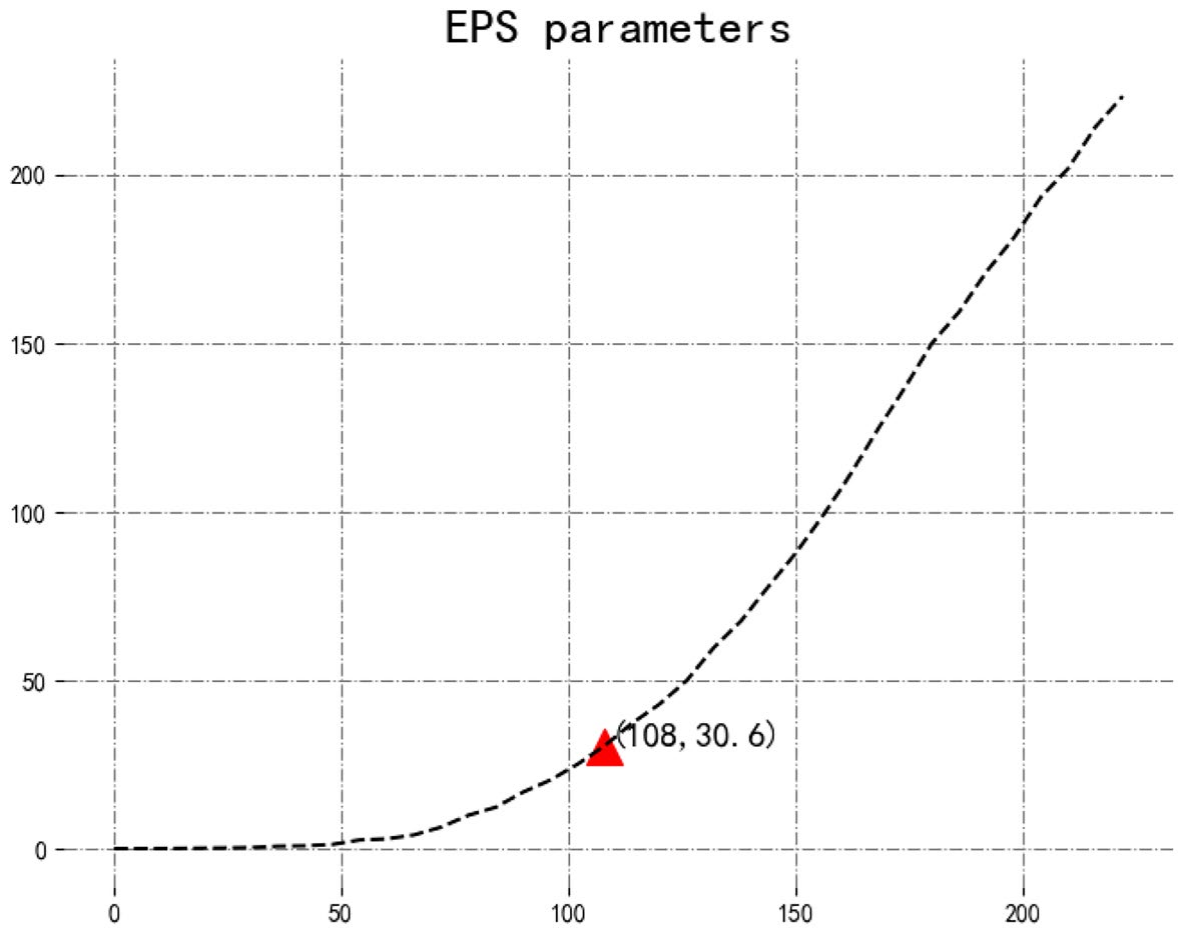
Sorted k-distance plots on pre-training data.

Currently, DBSCAN^30^ can find any shape clusters based on density. It has two key parameters eps and minPts. Moreover, Erich et al.^58^ proposed a method to find minPts based on the 2×dimensionality, and the appropriate value for eps based on the elbow in the k-distance. Since the dimension of clustering prototype vector is 2, the value of minPts is 4. Meanwhile, we use this method to get eps from Fig. 11.

From Fig. 11, it can be observed that the value of eps is 108. Note that Fig. 11 is an enlarged view of the eps part. Therefore, we can obtain the number of clusters.

Notable, the clustering prototype vector (Z_IT_) embedding space fuses the features of image- text. Our pre-train model Clus with 1.2million cluster number, which is much greater than the cluster number on ImageNet^54^. Due to the large quantity, we are unable to draw the distribution of all clusters by colors. Therefore, we select partial clusters to represent the distribution density by a heat map.

**Figure 12:**
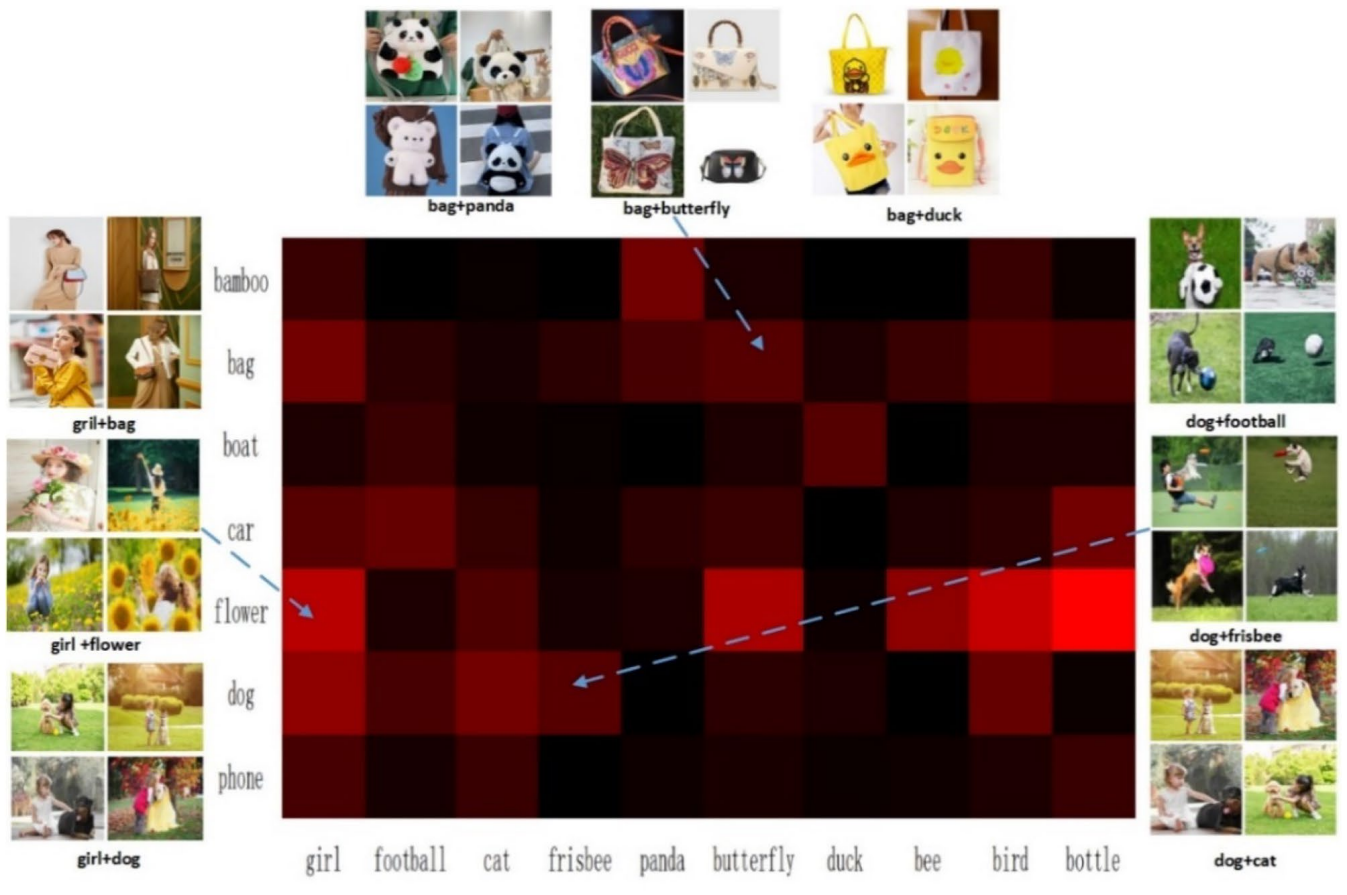
Partial clustering heat map. Red color is strong clustering density.

From Fig. 12, it can be observed that the image-text can be effectively fused and the clustering density matches the common sense of daily life. For example, we often see cat-dog, but we rarely see bamboo-frisbee. Therefore, we believe that this clustering is reasonable and valuable.

### Memory requirement

**Figure 13:**
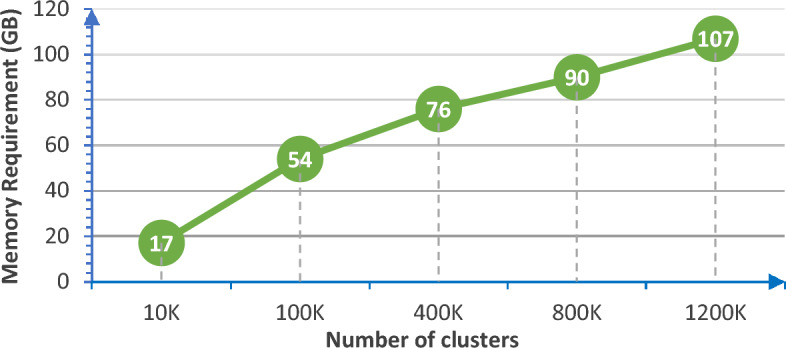
Memory requirement for scaling token length.

Given a lot of clusters and the use of LongNET for scaling token length, it is essential to discuss memory constraints associated with the model. The experimental results are as follow:

From Fig. 13, it can be observed that it is near a linear relationship between memory requirement and scaling token length. Specifically, memory requirement is acceptable. We analyze that this is because the dimension of the clustering prototype vector is 2 and LongNET saves memory^20^.

### Ablation studies

In order to verify the contribution of different components to the overall performance, we conduct detailed ablation studies. Due to the similar results on any dataset, our method is evaluated on object detection (i.e., COCO2017).

From Table 5, it can be observed that each component is beneficial for performance. Firstly, if we directly adopt image and text encoders from ALBEF, performance is reduced by 0.6. Thus, it is essential to select a good teacher network. Secondly, the co-attention mechanism is helpful, which is consistent with other methods (e.g., Coca, BEiT, etc.). Thirdly, when this study uses generic transformer layers (i.e., dim = R^197×768^), the result decreases by 1.4. This demonstrates that an increase in the number of active features can lead to a remarkable improvement in revenue. Similarly, if we give up clustering swap method, the ablation result is worse than replacing LongNET. We believe that the clustering center of image-text can better achieve feature semantic representation. Finally, the performance is unsatisfactory when we drop clustering swap prediction and LongNET, which further prove that the key ingredient of Clus is these two components.

**Table 5:** Ablation studies for Clus pre-training on object detection.

Models	COCO2017
Clus (our)	65.7
w/o—Distillation learning	65.1
w/o—Co-attention blocks	65.4
w/o—LongNET(Replace by normal transformer layer)	64.3
w/o—Clustering swap prediction	63.4
w/o—Clustering swap prediction and LongNET	62.2

### Discussion of experimental results

We conduct extensive experiments (i.e., Retrieval, VQA, NLVR2, Image Captioning, Object Detection, and Semantic Segmentation tasks) to verify the Clus. The experimental results demonstrate that the swap prediction method can help cluster centers learn appropriate image-text fusion features. In other words, a bit of improvement is great progress in performance. In addition, due to the difference among the three datasets in Nocaps, Clus only took the lead on the image captioning task in the in-domain dataset. Specifically, due to PaLI-17B^8^ with encoder–decoder architecture, it is an innate advantage in image captioning. However, Clus is already close to PaLI-17B^8^ in terms of metrics. In the future, we will increase the number of pre-training datasets and the number of clusters to improve the ability of image captioning generation.

## Conclusion

In this paper, we propose an image-text clustering swap prediction method to conduct multimodal fusion. At the same time, our model achieves SOTA performance on both downstream visual-text and vision tasks, which demonstrates that the increase of cluster number is beneficial. In particular, our method fills the gap of lack of clustering in multimodal methods and attempts to transfer generic models to practical tasks. Concurrently, Clus is with the same limitations as other methods, such as stopping knowledge updates after pre-training, and generating unfit advice or content, etc. Furthermore, Clus was pre-trained on partial open-source datasets with image-text, but we believe that other modality sets (e.g., audio, video, etc.) are very helpful for improving performance. Currently, due to extensive pre-trained data, Clus may generate offensive, biased content or be used maliciously. In order to mitigate negative impacts, we are attempting to address these issues by fine-tuning. Eventually, we attempt to find a balance between security and practicality.

In the following work, we will actively explore the application of generic models in industrial, electric power, medical, and other fields.

## Data Availability

All data needed to evaluate the conclusions in the paper are present in the paper. In addition, the data can be provided by corresponding authors.
